# Host-directed targeting of lincRNA-MIR99AHG suppresses intracellular growth of *Mycobacterium tuberculosis*

**DOI:** 10.1089/nat.2022.0009

**Published:** 2022-07-27

**Authors:** Lorna Gcanga, Ousman Tamgue, Mumin Ozturk, Shandre Pillay, Raygaana Jacobs, Julius Ebua Chia, Stanley Kimbung Mbandi, Malika Davids, Keertan Dheda, Sebastian Schmeier, Tanvir Alam, Sugata Roy, Harukazu Suzuki, Frank Brombacher, Reto Guler

**Affiliations:** 1 International Centre for Genetic Engineering and Biotechnology (ICGEB), Cape Town Component, Cape Town 7925, South Africa; 2 University of Cape Town, Institute of Infectious Diseases and Molecular Medicine (IDM), Department of Pathology, Division of Immunology and South African Medical Research Council (SAMRC) Immunology of Infectious Diseases, Faculty of Health Sciences, University of Cape Town, Cape Town 7925, South Africa; 3 Wellcome Centre for Infectious Diseases Research in Africa, Institute of Infectious Diseases and Molecular Medicine (IDM), Faculty of Health Sciences, University of Cape Town, Cape Town 7925, South Africa; 4 RIKEN Center for Integrative Medical Sciences, Yokohama, Japan; 5 Massey University, School of Natural and Computational Sciences, Auckland, New Zealand; 6 Department of Biochemistry, Faculty of Sciences, University of Douala, Douala, Cameroon; 7 South African Tuberculosis Vaccine Initiative, Institute of Infectious Disease and Molecular Medicine and Division of Immunology, Department Pathology, University of Cape Town, Cape Town 7925, South Africa; 8 Centre for Lung Infection and Immunology, Division of Pulmonology, Department of Medicine and UCT Lung Institute & South African MRC/UCT Centre for the Study of Antimicrobial Resistance, University of Cape Town, South Africa; 9 Faculty of Infectious and Tropical Diseases, Department of Infection Biology, London School of Hygiene and Tropical medicine, London, UK; 10 Information and Computing Technology Division, College of Science and Engineering, Hamad Bin Khalifa University, Doha 34110, Qatar

**Keywords:** *M. tuberculosis*, long non-coding RNAs, host-directed therapy, macrophages, inflammation

## Abstract

Tuberculosis (TB) caused by *Mycobacterium tuberculosis* (Mtb) kills 1.6 million people worldwide every year, and there is an urgent need for targeting host-pathogen interactions as a strategy to reduce mycobacterial resistance to current antimicrobials. Non-coding RNAs are emerging as important regulators of numerous biological processes and avenues for exploitation in host-directed therapeutics. Although long non-coding RNAs (lncRNAs) are abundantly expressed in immune cells, their functional role in gene regulation and bacterial infections remains under-studied. Here, we identify an immunoregulatory long intergenic non-coding RNA, lincRNA-MIR99AHG, which is upregulated in mouse and human macrophages upon IL-4/IL-13 stimulation and downregulated after clinical Mtb HN878 strain infection and in peripheral blood mononuclear cells (PBMC) from active TB patients. To evaluate the functional role of lincRNA-MIR99AHG, we employed antisense LNA GapmeR-mediated antisense oligonucleotide (ASO) lncRNA knockdown experiments. Knockdown of lincRNA-MIR99AHG with ASOs significantly reduced intracellular Mtb growth in mouse and human macrophages and reduced proinflammatory cytokine production. In addition, *in vivo* treatment of mice with MIR99AHG ASOs reduced the mycobacterial burden in the lung and spleen. Further, in macrophages, lincRNA-MIR99AHG is translocated to the nucleus and interacts with high affinity to hnRNPA2/B1 following IL-4/IL-13 stimulation and Mtb HN878 infection. Together, these findings identify lincRNA-MIR99AHG as a positive regulator of inflammation and macrophage polarization to promote Mtb growth and a possible target for adjunctive host-directed therapy against TB.

## Introduction

Tuberculosis (TB), caused by *Mycobacterium tuberculosis* (Mtb), is one of the leading infectious diseases in the world [1]. In 2018, TB killed 4110 people per day and 27400 people per day developed active TB globally [1]. Mtb is primarily transmitted by the respiratory route, and while it is able to cause disease in most organs, the most common involves the lungs [2]. New insights that target the host-pathogen interaction are crucial in the context of Mtb where there is a rise in antimicrobials resistance against the current 6 month regiment [3]. Mtb is able to utilize cellular host factors for its own survival and persistence [3]. Targeting these host factors exploited by Mtb could lead to a reduction in pathology, mycobacterial burden and possible latency [3]. Long non-coding RNAs (lncRNAs) have recently emerged as regulators of transcriptional programming to alter innate and adaptive immune responses [4].

LncRNAs are a family of non-coding RNAs (ncRNAs) mainly characterised as transcripts >200 nucleotides [5,6]. LncRNAs are known to act via post transcriptional mechanisms targeting the splicing, stability, or the translation of host mRNAs [7]. LncRNAs can act in *cis*- and *trans-* to regulate nearby genes or genes at other genomic locations [8–10]. Both *cis*- and *trans*-acting lncRNAs can activate or repress transcription through the recruitment of chromatin modifiers [8,9]. The role of lncRNAs has gained focus in recent years due to their importance in regulating macrophage biology [11–13]. For example, long intergenic non-coding RNA (lincRNA) *lincRNA-EPS* is an inhibitor of immune response gene (IRG) expression, acting as a regulatory checkpoint that is downregulated prior to inducible expression of IRGs [14]. The FANTOM 6 project has recently functionally annotated 285 human long non-coding RNAs through molecular phenotyping [15]. Many other lncRNAs including *lincRNA-Cox2* [16], *THRIL* [17], *Mirt2* [18] and *lnc13* [19] also regulate inflammatory genes in myeloid cells.

Recent studies have identified the functional role of lncRNAs during Mtb infection, which further implicated lncRNAs in immune responses [20–22]. A recently published review paper has summarised recent literature on differentially expressed lncRNAs and how they regulate the immune response to Mtb infection [23]. During active TB, *lncRNA-CD244* was highly expressed in CD244^+^CD8^+^ T-cells and acted as an epigenetic regulator of IFNγ and TNF-α expression by CD8^+^ T cells impacting CD8^+^ T-cell immunity against active Mtb infection [22]. *NEAT1* was highly expressed in PBMCs from patients with active TB when compared to healthy individuals [24]. Knockdown of *NEAT1* resulted in increased mycobacterial growth in infected THP1 cells [24]. The expression of lincRNA-Cox2 was induced in macrophages infected with Mtb and knockdown of lincRNA-Cox2 reduced NF-κB and STAT3 while increasing apoptosis [25]. *LncRNA-MEG3* was shown to contribute to mycobacteria clearance in macrophages infected with *Mycobacterium bovis* BCG via autophagy by targeting the mTOR and PI3K-AKT signalling pathways [21]. A microarray study examined expression profiles of lncRNAs in human macrophages infected with virulent H37Rv Mtb and avirulent H37Ra strains. Two lncRNAs *MIR3945HG V1* and *MIR3945HG V2* were identified as novel candidate diagnostic markers for tuberculosis [26]. Our understanding of the functional role of lncRNAs in M2 (IL-4/IL-13) polarized macrophages and during Mtb infection and their clinical relevance is still limited.

In this study, we identify lincRNA-MIR99AHG (hereon referred to as MIR99AHG) to be abundantly expressed in M2 (IL-4/IL-13) polarized mouse and human monocyte derived macrophages and downregulated in peripheral blood mononuclear cells (PBMC) from active TB patients and Mtb HN878 infected mouse and human macrophages. We demonstrate that MIR99AHG regulates inflammatory gene expression in macrophages stimulated with IL-4/IL-13 and infected with the clinical Mtb HN878 strain. Using antisense oligonucleotides (ASOs) lncRNA knockdown approaches, we show that knockdown of MIR99AHG in Mtb infected mice reduced mycobacterial burden in the lung and spleen. Mechanistically, we demonstrated in macrophages that MIR99AHG interacts with hnRNPA2/B1 in the nucleus following IL-4/IL-13 stimulation and Mtb HN878 infection. We propose a model whereby MIR99AHG regulates host-inflammatory response and promotes Mtb intracellular survival and persistence in macrophages.

## Material and Methods

### Ethics Statement

The BALB/c mouse strain was bred and housed in specific pathogen-free conditions and all animal procedures were performed in compliance with the standards of practice for laboratory animal procedures set by the Animal Research Ethics Committee, Faculty of Health Sciences, University of Cape Town, Cape Town, South Africa (Ethics approval Ref 015/040). The recruitment of healthy volunteers for this study was approved by the Human Ethics Committee, Faculty of Health Sciences, University of Cape Town, Cape Town (HREC Ref Number: 635/2015). Inclusion criteria were as follows: age 18-50 years, both sexes, no history of TB, no contact with TB patients, HIV negative, sputum smear-negative, non-smokers, no chronic alcoholism, normal chest x-rays, no chronic disease, not receiving immunosuppressive therapy, IGRA negative and absence of other pulmonary diseases. The participants who did not meet the above criteria, did not consent to signing the information consent form or to undertaking an HIV test were excluded from this study. The recruitment of active TB patients for this study was approved by the Human Ethics Committee, Faculty of Health Sciences, University of Cape Town, Cape Town (HREC Ref Number: 624/2015). Inclusion criteria were as follows: Informed consent, culture-positive individuals (aged 18-50 years), chest x-ray consistent with proven pulmonary TB and without prior TB treatment, sputum smear-positive and HIV negative.

### Mice

Wild-type BALB/c and IL-4Rα^-/-^ on a BALB/c background mouse (8-10 weeks) were housed under specific-pathogen-free conditions in individually ventilated cages.

### Generation of Bone Marrow-Derived Macrophages (BMDMs)

Bone marrow-derived macrophages were generated from 8-11 weeks old male BALB/c mice. The mice were sacrificed using halothane and death was confirmed by cervical dislocation, and their femur bones were flushed using plain Dulbecco’s modified Eagle’s medium (DMEM) (Gibco, Invitrogen Corporation, Carlsbad, CA, USA) to collect (centrifugation: 1200 rpm; 4°C; 10 minutes) bone marrow cells. The cells were seeded in sterile 100 mm CORNING plates (14 × 10^6^ cells/ml) and incubated (37°C; 5% CO_2_; 70% RH) for 10 days in PLUTZNIK media (DMEM containing 10% Foetal Calf Serum, 5% horse serum, 2mM L-glutamine, 1mM Na-pyruvate, 0.1mM 2-beta-Mercaptoethanol, 30% L929 cell-conditioned medium, 100U/ml penicillin G, 100μg/ml streptomycin) to allow for their differentiation into BMDMs. On day 10, the adherent cells were lifted using 4mg/ml Lidocaine EDTA solution and gentle scrapping, thereafter, washed twice with DMEM (containing 10% FCS, 100U/ml penicillin G, 100 μg/ml streptomycin) to remove residual M-CSF (present in the L929 conditioned medium). The BMDMs were seeded at 2-3 × 10^6^ cells/well, 5x10^5^cells/well and 1x10^5^ cells/well in 6, 24 and 96-well Nunc plates respectively and incubated (37°C; 5% CO_2_; 70% RH) overnight before performing downstream experiments.

### Generation of peripheral blood mononuclear cells (PBMCs) and monocyte-derived macrophages (MDMs)

PBMCs were isolated using Histopaque®-10771 (Sigma-Aldrich Biotechnology LP and Sigma-Aldrich Co., St Louis, MO) density gradient centrifugation. Blood was collected in VACUETTE® K3E blood collection tubes (Greiner Bio-one, GmbH, Germany), transferred to 50 ml Falcon tubes and diluted 1:2 with isotonic phosphate buffered saline (PBS) solution pH 7.4. 30 ml of diluted blood was transferred to 50 ml Leucosep™ 227290 tubes (Greiner Bio-one, GmbH, Germany) loaded beforehand with 15 ml Histopaque® solution as per the manufacturer instructions. The tubes were centrifuged at 1000 × g (2229 rpm) for 25 min (acceleration: 9; deceleration: 0) using an Eppendorf® 5810R centrifuge (A-4-81 Rotor, radius 18 cm). Plasma was discarded and buffy coat (lymphocytes / PBMCs) layer was harvested into a fresh 50 ml Falcon tube. The buffy coat was washed 3 times with 50 ml PBS solution pH 7.4 and spun at 250 × g for 10 min (acceleration 9, deceleration 5). PBMCs were re-suspended in 2 -5 ml complete growth medium (RPMI 1640 medium supplemented with 10% FCS, 2 mM L-glutamine and 1% penicillin G /streptomycin. All purchased from Life Technologies™, Carlsbad, CA, USA). PBMCs were plated at 1 × 10^5^ cells per 96-well tissue culture plates (Corning Costar®, Cambridge, MA) in complete growth medium for downstream bacterial burden experiments. For the generation of MDMs, PBMCs were plated in 6-/12-/96-well tissue culture plates (Corning Costar®, Cambridge, MA) at a density of 20/10/1 × 10^6^ cells per well respectively and incubated (37°C; 5% CO_2_; 70% RH) for 2 hours to allow monocytes to adhere. Non-adherent cells were discarded, and adherent monocytes were given a gentle wash with PBS then incubated in X-VIVO™ 15 serum free hematopoietic medium (supplemented with 1% penicillin G/streptomycin) for 7 days to allow for the differentiation of monocytes into MDMs. X-VIVO™ 15 serum free hematopoietic medium was changed on day 4. On day 7, X-VIVO™ 15 was removed, MDMs washed once with PBS and complete growth medium added for downstream experiments. MDMs purity was assessed by fluorescence-activated cell sorting (FACS) analysis using a PE–labeled anti-CD11b; PerCP–labeled anti-HLA-DR; FITC–labeled anti-CD14 and APC–labeled anti-CD3 monoclonal antibodies (All purchased from BD Biosciences™ CA, USA). MDMs purity exceeded 95%.

### ASO synthesis and purification

ASOs used in this study were synthesized by Exiqon ™ /Qiagen Germany. All ASOs including scrambled GapmeR negative control are 16 nucleotides in length and chemically modified with phosphorothioate backbone. The LNA GapmeRs were designed to provide potent and specific target knockdown. The primary design parameters include: 1) optimal target sequence accessibility to ensure high potency by selecting target sequences based on local secondary structure prediction, 2) antisense off-target evaluation by aligning GapmeR sequences against ENSMBL to allow selection of the most specific antisense LNA GapmeRs with minimal off-targets and 3) optimal ASO design including length, melting temperature, self complimentarity, LNA positions. The product sequences of ASOs used in the study are GAP-MIR99AHG 5’- CAGTTGCGTGGAGTAA-3’, GAP-MIR99AHG, 5’- CTAGCTTTGAAGTCGT-3’, GAP-hsMIR99AHG 5’-CAGTTGCGTGGAGTAA-3’ and scrambled GapmeR negative control 5’-AACACGTCTATACGC-3’. The ASOs were purified and analyzed by ESI-MS using anion-exchange HPLC, desalted and lyophilized as a sodium salt. The scrambled GapmeR negative control used in the study has the same chemical composition to target ASO-MIR99AHG. Purity and mass of oligonucleotides were confirmed by ESI-MS with more than 80% purity guaranteed. The ASOs were resuspended in TE buffer, sterile and filtered, and added to the cell culture medium.

### Transfection of BMDMs and MDMs by ASOs

Antisense LNA GapmeR targeting (Exiqon™/Qiagen, Germany) MIR99AHG were diluted in Opti-Mem medium (Life Technologies™) and mixed in a 1:1 ratio with Lipofectamine RNAiMAX 3000 (Life Technologies™). As a negative control, non-targeting scrambled LNA GapmeR (Exiqon™/ Qiagen, Germany) was transfected to the macrophages. For mouse and human macrophage cultures, two GapmeR MIR99AHG designs (5’-CAGTTGCGTGGAGTAA-3’ and 5’-CTAGCTTTGAAGTCGT-3’) were used to transfect murine macrophages and for the *in vivo* experiment in mice one GapmeR MIR99AHG design was used (5’-CAGTTGCGTGGAGTAA-3’). Cultured cells were transfected with 25 nM of GapmeR for 48 hours and the medium was replaced with DMEM or RPMI medium supplemented with 10% FCS for downstream experiments. *In vitro* dose response experiments were performed to optimize ASO transfections, investigating the effect of different concentrations of scrambled GapmeR negative control on BMDM cell viability. BMDMs treated with scrambled GapmeR negative control for 24, 48 or 72 hours at either 10nM or 50 nM did not cause any cell toxicity in cell culture plates seeded with 200’000 macrophages as confirmed by Cell Titre Blue assay (Figure S4A-C).

### Stimulation with *Mycobacterium tuberculosis, Mycobacterium bovis* BCG and *Leishmania mexicana* on BMDMs and MDMs

Macrophages were stimulated with recombinant mouse (100 units/ml) or recombinant human (10 ng/ml) IL-4, IL-13 or a combination of IL-4 and IL-13 (IL-4/IL-13) (BD Biosciences). At 24 hours post-stimulation macrophages were infected with a frozen stock of the clinical hypervirulent Mtb HN878 and mCherry expressing H37Rv strain, *Mycobacterium bovis* BCG and with *Leishmania mexicana* M379 strain at a multiplicity of infection (MOI) of 2 bacilli: 1 cell (2:1). Beads were used to disperse clumps of mycobacteria. After 4 hours, the supernatant was removed and fresh DMEM containing 10% FCS, 100 U/ml penicillin G, 100 μg/ml streptomycin, 10 ug/ml Gentamicin with or without stimulants was added to remove extracellular bacteria. Two hours later, the medium was replaced with DMEM containing 10% FCS with or without stimulants.

### RNA Extraction

Cells were lysed in Qiazol (Qiagen, Germany) at different timepoints post-transfection/-stimulation/-Mtb infection and lysates were stored at -80°C. Total RNA was isolated from the lysate using miRNeasy Mini kit (Qiagen, Germany) according to the manufacturer’s instructions. RNA quantity and purity were measured using the ND-1000 NanoDrop spectrophotometer (ThermoScientific, DE, USA).

### cDNA Synthesis and RT-qPCR

For protein coding gene and long non-coding RNA expression analysis, 100 ng total RNA was reverse-transcribed into cDNA using Transcriptor First Strand cDNA Synthesis Kit (Roche, Germany) according to the manufacturer’s instructions. Quantitative real-time PCR (RT-qPCR) was performed using LightCycler® 480 SYBR Green I Master (Roche, Germany) and gene-specific primers (IDT, CA, USA). Fold change in gene expression was calculated by the ΔΔCt method and normalized to Hprt1 which was used as internal control. The fold change expression was calculated using the ΔΔCt method to the housekeeping gene (HPRT) set to 1.

### Bacterial Burden Determination

At 24, 48 and 72 hours post-infection, supernatant was removed from Mtb-infected macrophages and stored at -80°C for analysis of cytokine production. Cells were lysed at 4, 48 aand 72 hours post infection in Triton X-100 and serial dilutions were plated on Middlebrook 7H10 agar plates and incubated for 15 days at 37°C in 5% CO_2_. Colony forming Units (CFUs) were enumerated to determine bacterial burden.

### Cytokine Production

Cytokine production was measured by enzyme-linked immunosorbent assay (ELISA) using ELISA development reagents (R&D systems, USA) at 24, 48 and 72 hours post Mtb. Culture supernatants were diluted 2, 3 and 6 folds. Data was acquired using Versamax™ Tunable microplate reader with Softmax Pro v6.3. (Avantor®, US).

### Griess reagent assay

Cultured supernatants were collected at 48 hours post Mtb to measure protein levels of nitrite, which correlates to nitric oxide production, by Griess reagents. Read out was acquired using Versama™ Tumble microplate reader with Softmax Pro v6.3 (Avantor®, US).

### RNA pull-down assay

Biotin-labelled lincRNA-MIR99AHG were transcribed with biotin RNA labelling mix and T7 RNA polymerase (Promega), treated with RNase-free DNase I (Promega), and purified using RNeasy Mini Kit (Qiagen). Biotin-labeled lincRNA-MIR99AHG was mixed and incubated with nuclear extracts of IL-4/IL-13 stimulated and Mtb HN878 infected BMDMs. Streptavidin-conjugated magnetic beads (Invitrogen) were added to each binding reaction and further incubated. Beads were washed thoroughly. Retrieved proteins were detected by mass spectrometry.

### RNA Immunoprecipitation (RIP)

HnRNPA2/B1 experiments were performed in nuclear extracts isolated from IL-4/IL-13 stimulated and Mtb HN878 infected BMDMs formaldehyde cross-linked conditions. Assays were performed as described in the Novex IP kit protocol (10007D) except that cells were crosslinked with 1% formaldehyde for 20 min. Nuclear extracts were precleared with protein G beads for 2 hours at 4°C and then incubated with control rabbit IgG or anti-hnRNPA2/B1 antibody (2 μg per sample) for 2 hours at RT. RNA was collected from 800 μl of each sample using TRIzol and treated with DNase to remove contaminating DNA. MIR99AHG levels were analysed by TR-qPCR. The remaining 200 μl of each sample was collected, boiled for 10min and subjected to Western blot analysis.

### Western blot

BMDMs were harvested and lysed in RIPA buffer supplemented with protease inhibitor (P8340, Sigma-Aldrich, US). Protein concentrations in cell lysates were measured using Pierce™ BCA protein assay kit (23225, Thermo Fischer Scientific, US), and the same amount of protein was loaded and separated on a polyacrylamide gel. Proteins were transferred to a nitrocellulose membrane. Membranes were blocked with 5% BSA solution (prepared in TBST) for 2 hours and probed with primary antibodies overnight in dilution buffer (TBST supplements with 1% BSA). The antibodies used in Western blots are: hnRNPA2/B1 (Santa cruz, sc-32316), hnRNPL (Abcam, ab6106), hnRNPK (Santa cruz, sc-28380), Cxadr (Santa cruz, sc-373791) and Gapdh (Santa Cruz, sc-365062). Membranes were probed with horseradish peroxidase-conjugated anti-mouse and anti-goat secondary antibodies (sc-2005 and sc-2020). Western blots were developed with LumiGlo Reserve™ chemiluminescent substrate kit (54-64-01, Sera care, Life Sciences, MA, US).

### *In vivo* LNA-GapmeR ASO treatment

18 Wild-type BALB/c mice randomly assigned were injected intraperitoneally with control (LNA GapmeR Negative Control A (10 mg/kg; Exiqon)) or ASO-MIR99AHG (10 mg/kg; Exiqon) for alternate days up to 14 days.

### Histology

Lungs sections were collected from euthanized mice and placed in 4% formaldehyde solution. Embedded sections were then stained with Hematoxylin and Eosin (H&E). The percentage of free alveolar spaces was defined as the open spaces in whole lung sections in relation to the total lung tissue area. Both free spaces and tissue areas were measured using the area measurement tool by the Nikon microscope imaging software NIS-elements and the % of alveolar spaces was calculated in Excel. A blinded quantification was performed to measure the percentage of MPO, CD3, iNOS and Caspase-3 using the Nikon microscope imaging software NIS-elements.

### Flow cytometry and lung myeloid cell sorting

Assessment of BMDM activation into M1 or M2 state, the extent of macrophage infection with mCherry expressing Mtb and assessment of apoptosis using Annexin V & 7-AAD staining was measured by flow cytometry. For surface staining of BMDM activation, cells were labelled with appropriate antibodies MHCII (M5/114.15.2), CD11b (M1/70), CD64 (X54-5/7.1), CD86 (GL1), CD80 (16-10A1) CD206 (C068C2), PD-L2 (TY25) purchased from BD Bioscience (Franklin Lakes, NJ, US) and eBioscience (San Diego, CA, US). After incubation, cells were resuspended in FACS buffer (0.1% BSA and 0.1%NaN_3_ in PBS) for acquisition. The acquisition was performed using BD LSRFortessa (BD Bioscience, NJ, US) and data were analysed using FlowJo software V10 (Treestar, Ashland, OR, US). Lung myeloid cell sorting was performed from the lungs of 3 weeks HN878 infected mice. Briefly, left lung and post caval lobes are mechanically minced before incubation in DMEM containing 0.18 mg/ml Collagenase Type I (Sigma), 0.02 mg/ml DNase I for 1 hr at 37 °C under rotation, followed by passing through 100 μm and 70 μm cell strainers sequentially. Red blood cells were lysed with lysis buffer (155 mM NH_4_Cl, 12 mM NaHCO_3_, 0.1 mM EDTA). Cells were then counted and subjected to antibody staining. 575V Fixable viability stain, CD64 (X54-5/7.1), CD11b (M1/70), CD11c (HL3), SiglecF (E5-2440), Ly6G (1A8) were used to stain lung myeloid cells for 20 min in 4 °C. Alveolar Macrophages, monocyte-derived DC and interstitial macrophages were sorted in complete media by using BD FACSAria Fusion. Cells were later spun at 400xg for 5 min and resuspended in Qiazol for downstream gene expression analysis. The gating strategies for flow cytometry experiments are provided in Figure S5C, S5D.

### RNA sequencing

Wild-type BMDMs were transfected with LNA GapmeRs and infected with Mtb HN878 with an MOI of 1:2 for 4 and 24 hours. RNA-seq libraries were prepared from total RNA and sequenced on Illumina HiSeq2000 as described [27].

### Mtb infection for determination of burdens in mice and *Listeria monocytogenes infection in mice for RT-qPCR analysis*

Anaesthetized mice were infected intranasally with 25 μl of viable HN878 Mtb bacilli into each nasal cavity with doses of 100 CFU/mouse for immune response analysis. Bacterial loads, histopathological and flow cytometry analyses in lungs of Mtb-infected mice were determined as previously described [28]. The lung weight index calculation was performed as a measure of inflammatory infiltration using: square root [(Lung weight in mg/Mouse weight in g)*10]/10. Briefly, aseptically harvested lungs and spleen were homogenized in 0.01% Tween-PBS and 10-fold dilutions were plated on 7H10 agar plates for the determinations of CFUs. Mice were infected intraperitoneally with *Listeria monocytogenes* (Lm) with a high-dose of 2 × 10^5^ CFU/mouse and liver and spleen were harvested for RT-qPCR analysis.

### Cell viability and apoptosis

Assessment of apoptosis was analysed by flow cytometry using the FITC Annexin V Apoptosis Detection Kit II (BD Bioscience). After incubation, cells were resuspended in FACS buffer for acquisition. Acquisition was performed using BD LSRFortessa (BD Bioscience, NJ, US) and data were analysed using FlowJo software (Treestar, Ashland, OR, US). Cell viability was assessed using Cell Titer Blue (Promega, US). After incubation, data was analysed using Versama™ Tumble microplate reader with Softmax Pro v6.3 (Avantor®, US). Apoptosis of macrophages *in vitro* was assessed with the fluorescent *in situ* TUNEL assay according to the manufacturer’s specifications (*In Situ* Cell Death Detection Kit, Roche). BMDMs were incubated for 1 hour with 4% paraformaldehyde at RT. Macrophages were washed once with 1X PBS and incubated for 2 min on ice with a permeabilization solution (0.1% Triton X-100) and washed twice with 1X PBS. Macrophages were labelled with the TUNEL reaction mixture for 1 hour at 37°C then analysed with fluorescent microscopy.

### Statistical analysis

All data were analysed using GraphPad Prism v6.0, a Student’s t-test (two-tailed with unequal variance) or unless otherwise stated in Figure legends. Means are shown as SD., *P < 0.05, **P < 0.01, ***P< 0.001 and ****P < 0.0001 respectively.

## Results

### MIR99AHG is upregulated in M2 (IL-4/IL-13) murine and human macrophages, downregulated following Mtb HN878 infection and dependent on the IL-4Rα pathway

**Figure 1:**
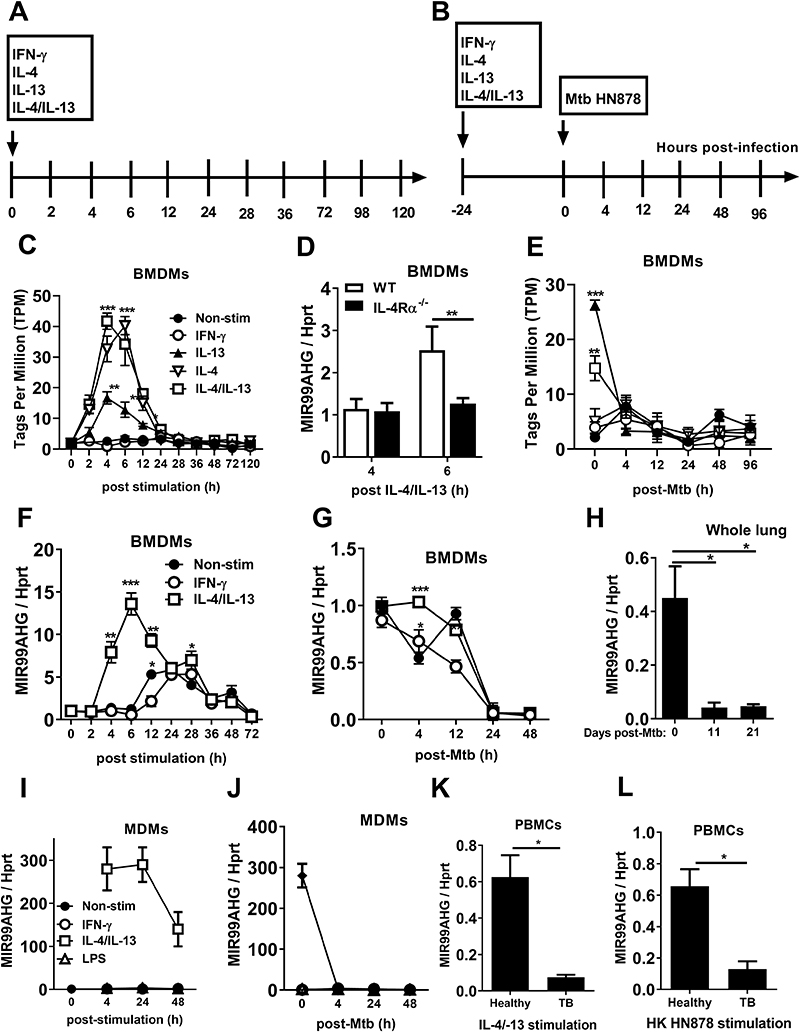
MIR99AHG is upregulated in M2 (IL-4/IL-13) in murine and human macrophages, downregulated following Mtb HN878 infection and dependent on the IL-4Rα pathway. Bone marrow cells were differentiated for 10 days into BMDMs and stimulated with IFNγ or IL-4, IL-13, IL-4/IL-13. At 24 hours post-stimulation, BMDMs were infected with Mtb HN878 for 4, 24 and 48 hours. RNA was extracted from lysed cells at different time points post-stimulation and post-Mtb HN878 infection. (**A, B**) Timeline of mouse macrophage cytokine stimulation and Mtb HN878 infection. (**C, E**) CAGE analysis of MIR99AHG TPM kinetic expression in cytokine stimulated and Mtb HN878 infected BMDMs. Data are representative of three pooled independent experiments. (**D**) MIR99AHG mRNA expression by RT-qPCR from IL-4Rα^-/-^ BMDMs stimulated with IL-4/IL-13 for 4 and 6 hours. Data are representative of three pooled independent experiments. (**F, G**) RT-qPCR analysis of MIR99AHG kinetic mRNA expression in cytokine stimulated and Mtb HN878infected BMDMs. Data are representative of three pooled independent experiments. (**H**) RT-qPCR analysis of MIR99AHG mRNA expression on whole lung from wild-type BALB/c mice infected with Mtb HN878 (100 CFU/mouse) for 11 and 21 days. Data are representative of three independent experiments. (**I**) RT-qPCR analysis of MIR99AHG kinetic expression in cytokine stimulated human MDMs. Data are representative of three pooled independent experiments. (**J**) RT-qPCR analysis of MIR99AHG kinetic expression in Mtb HN878 infected human MDMs. (**K, L**) Human MIR99AHG mRNA expression by RT-qPCR following IL-4/IL-13 and heat killed Mtb HN878 at 4 hours post stimulation in PBMCs isolated from healthy and active TB patients. The fold change in gene expression was determined by RT-qPCR and was normalised to the housekeeping gene HPRT.Data are expressed as mean ± SD of triplicates. Data are representative of three independent experiments. P values represented as, *P < 0.05, ** *P* < 0.01 and ***P < 0.001; Two-way ANOVA (C-F) and Bonferroni post-hoc test and Student’s *t*-test (F).

We previously utilized deep CAGE (Cap Analysis of Gene Expression) transcriptomics to define the transcriptome of M1 (IFN-γ) or M2 (IL-4, IL-13, IL-4/IL-13) activated bone-marrow-derived macrophages (BMDMs) [12,29,30] and during Mtb HN878 infection [31,32]. Using this approach (Figure 1A, B), we identified several differentially expressed lncRNAs with MIR99AHG being highly expressed in M2 (IL-4/IL-13) stimulated macrophages (Figure 1C). At 4 hours post stimulation, IL-4/IL-13 upregulated 40-fold the expression CAGE tags per million (TPM) of MIR99AHG when compared to the unstimulated macrophages. This upregulation was mediated by IL-4/IL-13 signalling via the common IL-4 receptor alpha chain, since in IL-4Rα^-/-^ BMDMs, we did not induce the expression of MIR99AHG to the same levels as wild type (WT) (Figure 1D). In contrast, Mtb HN878 infection repressed MIR99AHG expression in a time dependent manner (Figure 1E). The CAGE TPM expression of MIR99AHG was validated by RT-qPCR in cytokine-stimulated and Mtb HN878 infected macrophages (Figure 1F, G). In addition, MIR99AHG was downregulated in mice infected with Mtb HN878 at 11 and 21 days post infection (Figure 1H). As in BMDMs, human MIR99AHG expression in monocyte derived macrophages (MDMs) was upregulated by IL-4/IL-13 stimulation and downregulated by Mtb HN878 infection (Figure 1I, J). We re-stimulated PBMCs from healthy and active TB patients with IL-4/IL-13 and heat-killed Mtb HN878 for 4 hours and observed reduced mRNA expression of MIR99AHG (Figure 1K, L). Altogether, these data show that MIR99AHG expression varies according to the macrophage polarization state, in IL-4/IL-13 polarized macrophages MIR99AHG expression was induced while the expression of MIR99AHG was downregulated following Mtb HN878 infection and in active TB patients.

To confirm whether other Mtb strains and other species of mycobacterium can regulate MIR99AHG expression, BMDMs were polarized with IL-4/IL-13 and then infected with the laboratory H37Rv, H37Ra strain and *Mycobacterium bovis* BCG to measure MIR99AHG mRNA expression by RT-qPCR (Figure S1A,-C). Similarly, to Mtb HN878, H37Rv and H37Ra infection resulted in the downregulation of MIR99AHG mRNA expression at 4 hours post infection (Figure S1A, B). In contrast, *Mycobacterium bovis* BCG infection resulted in the upregulation of MIR99AHG (Figure S1C). MIR99AHG downregulation was also observed in BMDMs pre-stimulated with IL-4/IL-13 and infected with *Leishmania mexicana* (Figure S1D) suggesting that MIR99AHG is downregulated by other intracellular pathogens that infect macrophages. To confirm whether other intracellular pathogens could reduce MIR99AHG expression, mice were infected with *Listeria monocytogenes* to determine MIR99AHG mRNA expression by RT-qPCR. Whole spleen and liver of mice infected *with Listeria monocytogenes* significantly displayed reduced mRNA expression of MIR99AHG at 2 days post infection (Figure S1 E, F).

### Mtb HN878-induced suppression of MIR99AHG is mediated by NF-κB but not p38 signalling pathway and MIR99AHG expression is differentially regulated by TLR agonists

**Figure 2:**
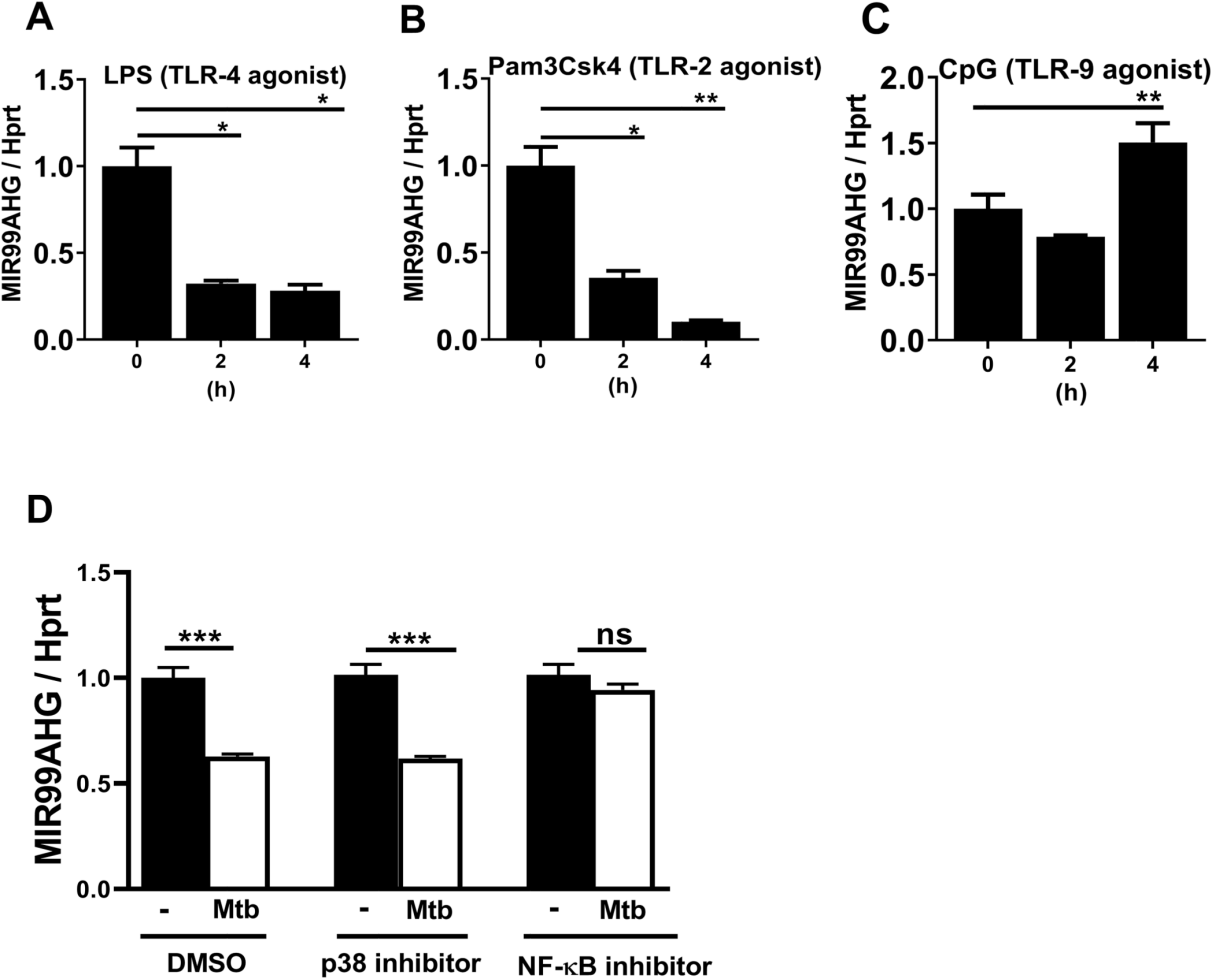
MIR99AHG downregulation by Mtb in murine macropahages is mediated by the NF-κB but not the p38 signalling pathway and MIR99AHG expression is differentially regulated by TLR agonists. (**A-C**) RT-qPCR of MIR99AHG mRNA expression in bone marrow-derived macrophages (BMDMs) stimulated with TLR-9 agonist CpG (500 nM), TLR-4 agonist LPS (100 ng/ml) and TLR-2 agonist Pam3Csk4 (100 ng/ml). Data are representative of three pooled independent experiments. (**D**) BMDMs were pre-treated for one hour with selective pharmacological inhibitors for NF-κB (Bay11-7082; 10 μM), p38 (SB203580; 5 μM) and infected with Mtb HN878 for 4h. Expression of MIR99AHG mRNA was detected by RT-qPCR. NS= not significant. Data are representative of three independent experiments. Data are expressed as mean ± SD of triplicates. P values represented as, *P < 0.05, **P < 0.01, and ***P < 0.001, Student’s *t*-test.

We employed RT-qPCR to examine the kinetics of MIR99AHG expression in BMDMs exposed to TLR ligands including CpG (TLR-9), LPS (TLR-4) and Pam3CSK4 (TLR-2/1). The response of mRNA expression of MIR99AHG was significantly downregulated in BMDMs exposed to LPS and Pam3CSK4 in a time dependent manner but significantly upregulated in BMDMs exposed CpG at 4 hours post stimulation (Figure 2A-C). Selective pharmacological inhibitors for NF-κB (Bay11-7082) and p38 (SB203580) were employed to assess the functional relevance of these signalling pathways on regulating MIR99AHG expression. The p38 inhibitor (SB203580) did not restore the downregulation of MIR99AHG expression by Mtb HN878 infection (Figure 2D). In contrast, the NF-kB inhibitor (Bay11-7082) restored the Mtb HN878-induced downregulation of MIR99AHG expression (Figure 2D). Collectively, these results indicate the NF-κB but not the p38 signalling pathway is responsible for the Mtb-mediated downregulation of MIR99AHG.

### Knockdown of MIR99AHG by ASOs reduces intracellular Mtb growth, necrosis, pro-inflammatory cytokines and increases early apoptosis in murine macrophages

**Figure 3:**
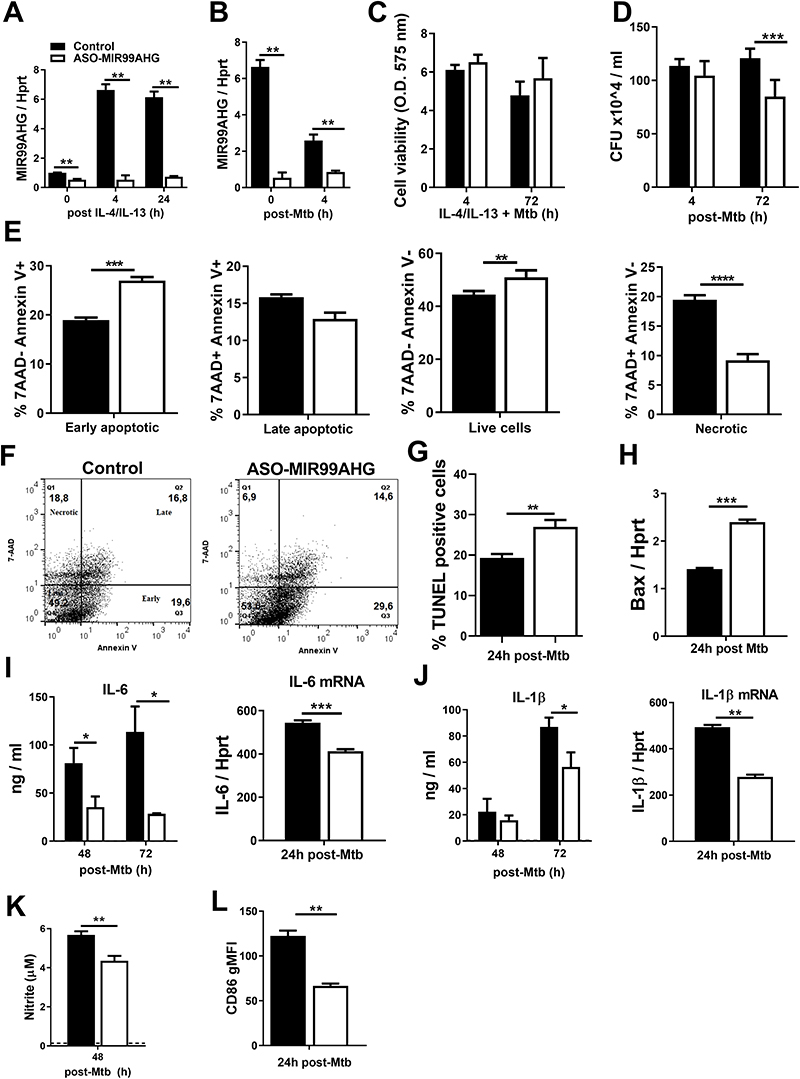
Knockdown of MIR99AHG by ASOs reduces intracellular Mtb growth, necrosis, pro-inflammatory cytokines and increases early apoptosis in murine macrophages. BMDMs were ASO transfected with locked nucleic acid control and ASO-MIR99AHG, stimulated with IL-4/IL-13 for 4 hours and infected with Mtb HN878. (**A**) MIR99AHG mRNA expression by RT-qPCR in ASO-treated BMDMs post IL-4/IL-13 stimulation (**B**) and post Mtb HN878 infection. Data are representative of three independent experiments. (**C**) Cell viability was measured in BMDMs at 4 and 72 hours post Mtb HN878 infection by Cell Titer Blue. Data are representative of three independent experiments. (**D**) BMDMs were ASO transfected with control and ASO-MIR99AHG, pre-stimulated with IL-4/IL-13 for 4 hours and infected with Mtb HN878. BMDMs were lysed at 4h uptake and 72h post-Mtb HN878 infection to measure mycobacterial growth by CFU counting. Data are representative of three pooled independent experiments. (**E-H**) BMDMs were antisense oligonucleotides (ASOs) treated with control and ASO-MIR99AHG for 48 hours, then stimulated with IL-4/IL-13 for 4 hours and infected with Mtb HN878 for 24 hours to measure early apoptotic, late apoptotic, necrotic and live cells with Annexin V and 7-AAD staining by flow cytometry. Data are representative of three independent experiments. (**F**) Representative flow dot plots by flow cytometry. (**G**) At 24 hours post Mtb HN878 infection, BMDMs were labelled with the TUNEL reaction mixture and the percentages of TUNEL positive apoptotic cells analysed by fluorescent microscopy. Data are representative of two independent experiments. (**H**) BMDMs were ASO transfected with control and ASO-MIR99AHG, pre-stimulated with IL-4/IL-13 for 4 hours, infected with Mtb HN878 and RNA collected at 24 hours post Mtb. Bax mRNA expression by RT-qPCR in BMDMs infected with Mtb HN878 at 24 hours post infection. Data are representative of three independent experiments. (**I, J**) Protein and mRNA cytokine levels of IL-6 and IL-1β measured by ELISA and RT-qPCR. Data are representative of three independent experiments. (**K, L**) Nitrite production measured by Griess reagent assay and CD86 gMFI by flow cytometry. Data are representative of three independent experiments. Data are expressed as mean ± SD of triplicates (A, B, C, E, H, K, L) and quadruplets (D, I, J). P values represented as, *P < 0.05, **P < 0.01, ***P < 0.001, and ****P < 0.0001; Student’s *t*-test.

To examine the functional role of MIR99AHG in regulating the intracellular growth of Mtb in murine macrophages, we performed lncRNA loss-of-function experiments using Locked Nucleic Acid (LNA) chemically engineered GapmeR antisense oligonucleotides (ASOs). We first measured the knockdown efficiency of ASOs in M2 (IL-4/IL-13) polarized macrophages and identified a 92% reduction of MIR99AHG expression at 4 hours post IL-4/IL-13 stimulation measured by RT-qPCR (Figure 3A). We also identified a 73% reduction of MIR99AHG expression at 24 hours post IL-4/IL-13 stimulation (Figure 3A) and a 69% reduction at 4 hours post Mtb HN878 infection (Figure 3B). ASO treatment for MIR99AHG did not result in any macrophage cell toxicity during IL-4/IL-13 stimulation and Mtb HN878 infection as we identified stable cell viability measured by the Cell Titre Blue assay (Figure 3C).

To examine the functional role of MIR99AHG during Mtb HN878 infection, we inhibited MIR99AHG using ASOs and analysed its effect on the intracellular growth of Mtb in macrophages by colony forming units (CFU) assays. Knockdown of MIR99AHG by ASOs significantly reduced the intracellular Mtb growth in IL-4/IL-13 stimulated macrophages at 72 hours post Mtb HN878 infection (Figure 3D) but did not change the phagocytic ability of macropahges to uptake Mtb at 4 hours post infection. This non significant change in CFU at 4 hours post infection between the control and ASO-MIR99AHG treated macrophages is an important measure because it rules out impairment in the phagocytic uptake of Mtb by the macrophage. Knockdown of MIR99AHG by ASOs led to an increased early apoptosis and reduced necrosis measured by Annexin V and 7AAD staining in IL-4/IL-13 stimulated BMDMs following Mtb HN878 infection, suggesting a possible mechanism for the reduced intracellular Mtb growth (Figure 3E, F and Figure S2B). Knockdown of MIR99AHG by ASOs led to increased apoptosis as measured by staining for DNA strand breakage using the TUNEL assay (Figure 3G) at 24 hours post Mtb infection. Using RT-qPCR, apoptosis was further confirmed at 24 hours post Mtb HN878 infection by measuring the mRNA expression of Bax, a pro-apoptotic marker, which was significantly increased in ASO MIR99AHG treated BMDMs (Figure 3H). We chose to measure early apotosis and Baxat 24 hours post Mtb infection as more than 80% of macrophages have phagocytosed mycobacteria at this time point.

To further characterize the functional role of MIR99AHG in modulating pro-inflammatory cytokine responses, we collected supernatant and RNA from ASO transfected BMDMs and performed ELISA and RT-qPCR. Protein and mRNA levels of IL-6 (Figure 3I) and IL-1β (Figure 3J) were significantly reduced in ASO MIR99AHG treated BMDMs when compared to controls following IL-4/IL-13 stimulation and Mtb HN878 infection. We measured nitrite production by Griess reagent assay from ASO transfected BMDMs, stimulated with IL-4/IL-13 and infected with Mtb HN878. Knockdown of MIR99AHG by ASOs significantly reduced nitrite production compared to control in IL-4/IL-13 stimulated and Mtb HN878 infected macrophages (Figure 3K). Furthermore, the silencing of MIR99AHG by ASOs significantly decreased CD86 gMFI, a known marker for classically activated macrophages (Figure 3L). These data suggest that Mtb regulates inflammatory host responses by targeting MIR99AHG to promote intracellular growth in macrophages.

### Knockdown of MIR99AHG by ASOs reduces intracellular Mtb growth, IL-6 and Bax in Mtb-infected human macrophages

**Figure 4:**
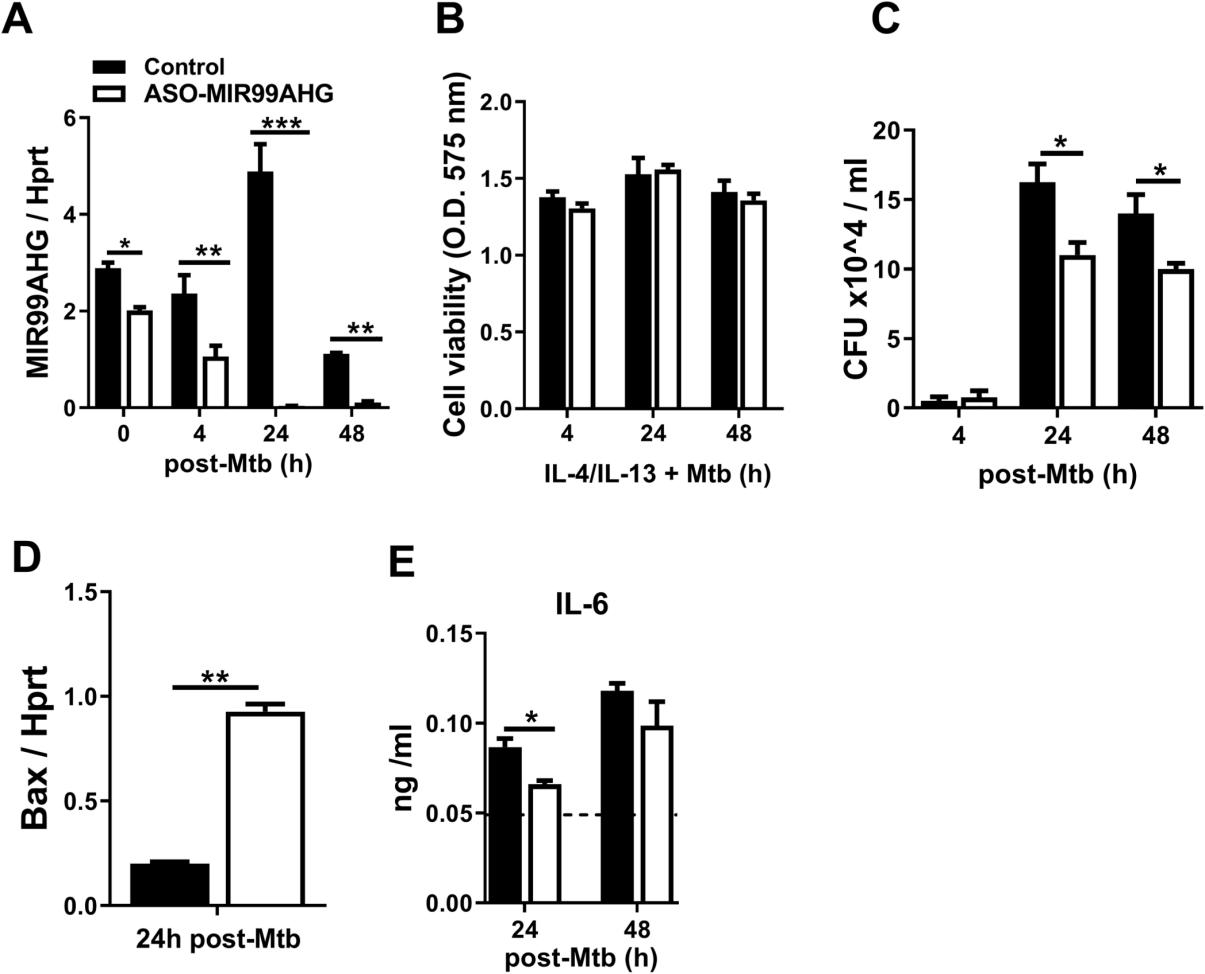
Knockdown of MIR99AHG by ASOs reduces intracellular Mtb growth, IL-6 and Bax in Mtb-infected human macrophages (**A**) MDMs were ASOs transfected with control and ASO-MIR99AHG, pre-stimulated with IL-4/IL-13 and infected with Mtb HN878. MIR99AHG mRNA expression by RT-qPCR in ASO-treated MDMs. Data are representative of three independent experiments. (**B**) Cell viability of ASOs transfected human MDMs pre-stimulated with IL-4/IL-13 for 4 hours and infected with Mtb HN878. Data are representative of three independent experiments. (**C**) MDMs were ASO transfected with control and ASO-MIR99AHG, pre-stimulated with IL-4/IL-13 for 4 hours and infected with Mtb HN878. Cells were lysed at 4h for uptake and 24 and 48h post-Mtb HN878 infection to measure bacterial growth by CFU counting. Data are representative of three pooled independent experiments. (**D**) Bax mRNA expression by RT-qPCR in human MDMs infected with Mtb HN878. Data are representative of three independent experiments. (**E**) IL-6 production in human MDMs pre-stimulated with IL-/IL-13 for 4 hours and infected with Mtb HN878 measured by ELISA. Data are representative of three independent experiments. Data are expressed as mean ± SD of triplicates. P values represented as, *P < 0.05, **P < 0.01 and ***P < 0.001, Student’s *t*-test.

To confirm the results observed in mouse macrophages, we examined the expression of MIR99AHG in MDMs. We employed RT-qPCR to examine the kinetics of MIR99AHG expression on ASO transfected human MDMs pre-stimulated with IL-4/IL-13 and infected with Mtb HN878. The expression of human MIR99AHG was downregulated in IL-4/IL-13 polarized macrophages following Mtb HN878 infection (Figure 4A). Human MDMs were viable following ASO treatment as confirmed by Cell Titre-blue (Figure 4B). Knockdown of MIR99AHG by ASOs significantly reduced Mtb intracellular growth in human MDMs (Figure 4C). The mRNA levels of the pro-apoptotic marker Bax were significantly increased in MIR99AHG ASO treated human MDM at 24 hours post Mtb HN878 infection (Figure 4D). In IL-4/IL-13 stimulated and Mtb HN878 infected human MDMs, the knockdown of MIR99AHG by ASOs resulted in significantly reduced protein levels of IL-6 (Figure 4E). These results demonstrate that MIR99AHG is targeted by Mtb to promote intracellular growth in human macropahes.

### *In vivo* ASO knockdown of MIR99AHG reduces mycobacterial burden in mice and pro-inflammatory responses in lung macrophages

**Figure 5:**
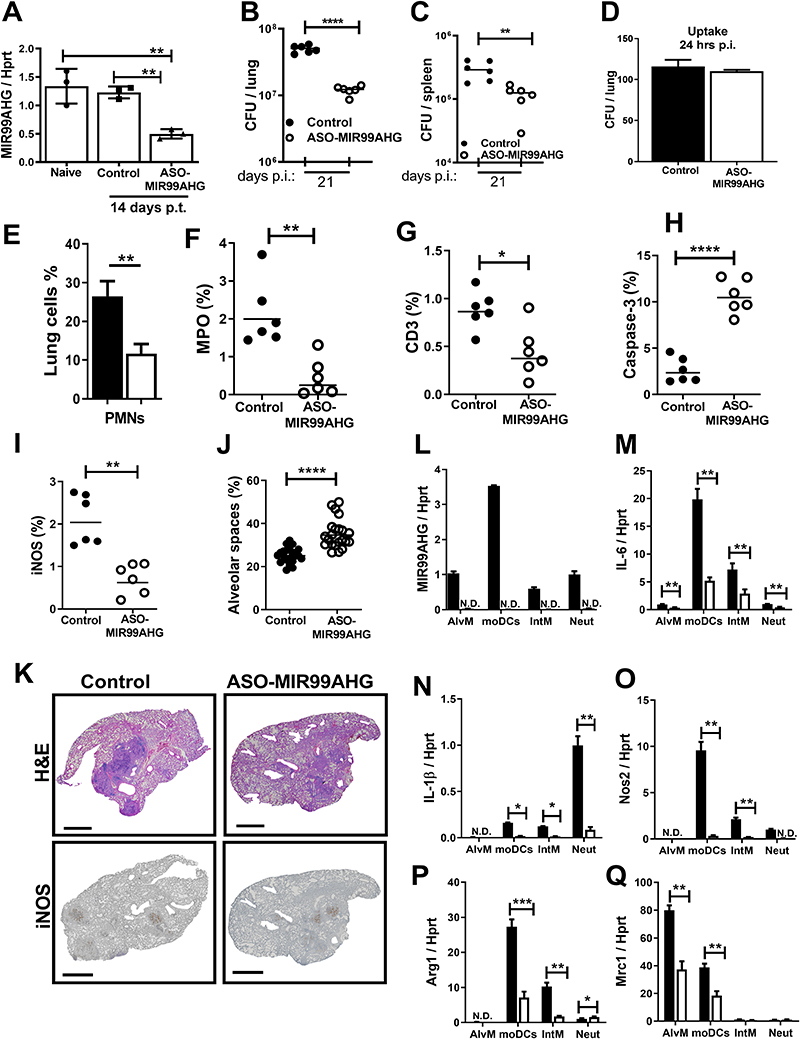
*In vivo* ASO knockdown of MIR99AHG reduces mycobacterial burden in mice and pro-inflammatory responses in lung macrophages BALB/c mice (n=6/mice/group) were ASO treated with 10 mg/kg locked nucleic acid control and ASO-MIR99AHG. (**A**) MIR99AHG mRNA expression by RT-qPCR at 14 days post ASO treatment. (**B, C**) Mycobacterial burden of Mtb HN878-infected mice are shown with indicated CFU in lung and spleen at 3 weeks post infection. (**D**) Mtb HN878 infected mice were sacrificed at 24 hours post infection to determine the CFU lung uptake (n = 3 mice/group). (**E**) Percentage of lung neutrophils at 3 weeks post infection quantified by flow cytometry. (**F-I**) The percentage of positive MPO, CD3, Caspase-3 and iNOS staining was quantified from 2 deep cut lung sections per mice at 3 weeks post Mtb HN878 infection (30 μm apart). (**J**) Alveolar spaces at 3 weeks post infection were quantified from 4 deep cut H&E lung sections per mice (30μm apart). (**K**) Representative histopathology sections (x2 magnification) at 3 weeks post infection for H&E and iNOS (scale bar=1000 μm). (**L-Q**) At 3 weeks post infection, CD64^+^CD11c^+^SiglecF^+^ alveolar macrophages (AlvM), CD11b^+^CD11c^+^CD64^+^ (moDCs), CD64^+^CD11b^+^CD11c^-^SiglecF^-^ interstitial recruited macrophages (IntM) and CD11b^+^LY6G^+^ neutrophils were sorted by flow cytometry to determine lung mRNA expression of MIR99AHG, *IL6, IL1b Nos2*, Arg1 and Mrc1 by RT-qPCR. N.D.= not detected. Data are expressed as mean ± SD. P values represented as, *P < 0.05, ** *P* < 0.01, and **** *P* < 0.0001; Student’s *t*-test.

Targeting of non-coding RNAs to promote host innate defence is a promising strategy in the development of novel therapeutic interventions against TB [3,33]. To investigate whether the *in vitro* beneficial effects of MIR99AHG suppression in reducing intracellular Mtb growth and pro-inflammatory cytokines (Figure 2) could be translated into an *in vivo* host-directed therapy (HDT), we used antisense LNA-modified GapmeR ASOs to inhibit MIR99AHG in a mouse Mtb infection model. First, the mice were subcutaneously treated with ASOs for MIR99AHG or control (Antisense LNA GapmeR Negative control A) at 10 mg/kg for alternate days up to day 14. Mice were then infected with the hypervirulent clinical Mtb HN878 strain (100 CFU/mouse) intranasally for 21 days. RT-qPCR results showed that ASO treatment drastically suppressed MIR99AHG expression in lung tissues with a knockdown efficiency of 73% (Figure 5A). Mice treated with MIR99AHG ASOs showed significantly reduced mycobacterial load in the lungs and in the spleen when compared to the control mice (Figure 5B and C). There was no difference in the Mtb uptake into the lungs measured by colony forming units (CFU) assay at 1 day post infection between the control mice and MIR99AHG ASO-treated mice (Figure 5D).

At 3 weeks post infection, there was reduced percentage of neutrophils in the lungs in the ASO-MIR99AHG treated mice compared to the control mice (Figure 5E). Myeloperoxidase (MPO) assay validated reduced neutrophil influx into lungs in MIR99AHG ASO-treated mice (Figure 5F). T cell recruitment, measured by CD3 staining, was significantly reduced in MIR99AHG ASO-treated mice (Figure 5G). Caspase-3, a marker for apoptosis, was significantly increased in MIR99AHG ASO-treated mice compared to the control group (Figure 5H). Staining of lung sections with inducible nitric oxide synthase (iNOS), the enzyme responsible for producing the anti-mycobacterial effector molecule nitric oxide (NO), was significantly reduced in MIR99AHG ASO-treated mice when compared to the control group (Figure 5I, K) suggesting that the observed anti-mycobacterial effector functions were independent of iNOS. Alveolar spaces measured on H&E lung sections, were significantly increased in MIR99AHG ASO-treated mice as compared to the control mice at 3 weeks post Mtb HN878 infection, suggesting reduced pulmonary lesion sizes following MIR99AHG inhibition (Figure 5J, K).

To better define inflammatory responses in the lungs following MIR99AHG ASO treatment, myeloid cell populations were sorted with flow cytometry to measure mRNA expression by RT-qPCR at 3 weeks post Mtb HN878 infection. MIR99AHG expression was expressed in alveolar macrophages (AlvM), monocyte-derived dendritic cells (moDCs), interstitial recruited macrophage (IntM) and neutrophils (Neut) from Mtb HN878infected control mice but absent in MIR99AHG ASO-treated mice (Figure 5L). Notably, MIR99AHG ASO treatment displayed significantly reduced levels of pro-inflammatory cytokines, such as *il6* and *il1b* when compared to controls mainly in moDCs, IntM and Neut cell populations (Figure 5M, N). In addition, nitric oxide synthase (NOS2), Arg1 and Mrc1 mRNA expression was significantly reduced in moDCs, IntM and AlvM from Mtb HN878 infected MIR99AHG ASO-treated mice (Figure 5O-Q). T lymphocyte population in the lungs were not affected by the knockdown of MIR99AHG except for increased percentage of CD4^+^ T_effector_ cells (Figure S3A-D; Figure S2A). Taken together, these results suggest that MIR99AHG contributes to mycobacterial growth, increases pulmonary histopathology and enhances pro-inflammatory responses in lung macrophages.

### MIR99AHG is translocated from the cytoplasm to the nucleus following IL-4/IL-13 stimulation and interacts with hnRNPA2/B1

**Figure 6:**
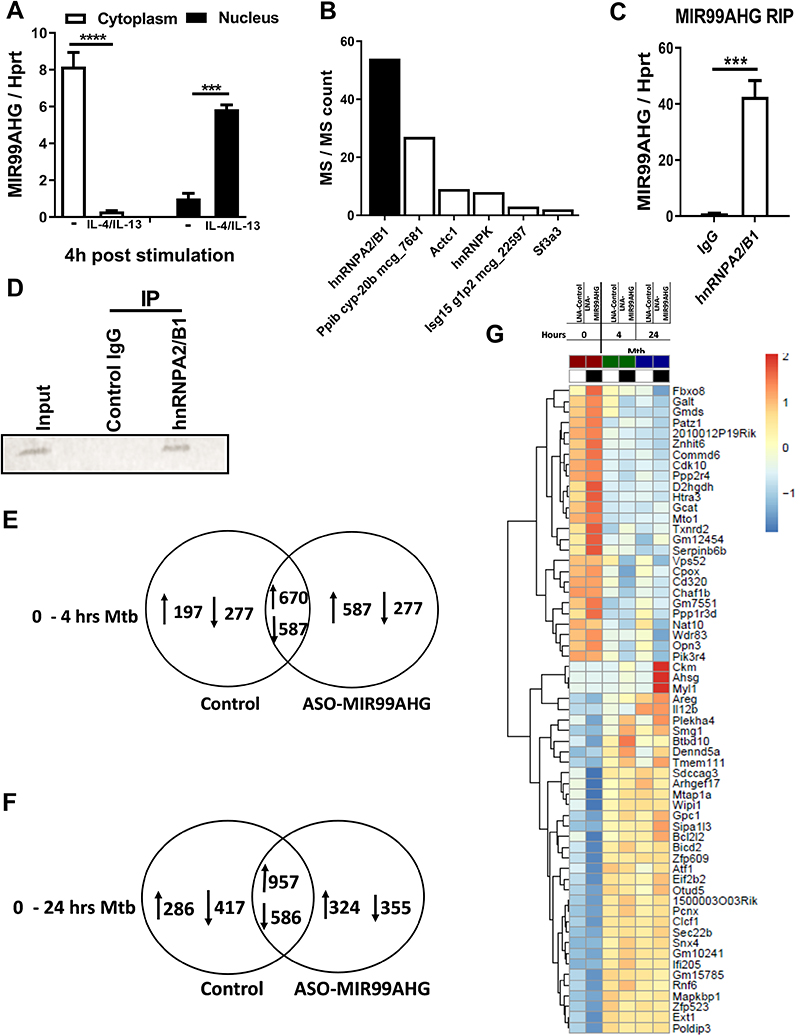
MIR99AHG is translocated from the cytoplasm to the nucleus following IL-4/IL-13 stimulation and interacts with hnRNPA2/B1 in murine macrophages. (**A**) RT-qPCR analysis of RNAs purified from nuclear and cytoplasm compartments in BMDMs stimulated with IL-4/IL-13 for 4h. Data are representative of three independent experiments. (**B**) The 6 most enriched proteins associated with MIR99AHG identified by affinity purification and mass spectrometry in ASO treated BMDMs pre-stimulated with IL-4/IL-13 for 4 hours and infected with Mtb HN878 for 24 hours. Data are representative of three independent experiments. (**C**) hnRNPA2/B1 RIP followed by mRNA expression of MIR99AHG by RT-qPCR in anti-hnRNPA2/B1 antibody or control IgG immunoprecipitates from nuclear lysates of macrophages pre-stimulated with IL-4/IL-13 for 4 hours and infected with Mtb HN878 for 24 hours. (**D**) Western blot of hnRNPA2/B1 in Input, anti-hnRNPA2/B1 antibody and control IgG immunoprecipitates from nuclear lysates of macrophages pre-stimulated with IL-4/IL-13 for 4 hours and infected with Mtb HN878 for 24 hours. (**E-G**) BMDMs were antisense oligonucleotide (ASO) transfected with locked nucleic acid control and ASO-MIR99AHG for 48 hours, and infected with Mtb HN878 for 4 and 24 hours. (E, F) Venn diagrams displaying up and down regulated genes between control ASO-MIR99AHG at 4 and 24 hours post Mtb infection. Data are representative of three independent experiments. (**G**) Heatmap representative of genes commonly upregulated and downregulated at 4 and 24 hours post Mtb infection. Each row (gene) was centered on the mean expression value across all samples. Data are representative of three independent experiments. Data shown is mean of three replicates. Data are expressed as mean ± SD (A, C) of triplicates. P values represented as, *** *P* < 0.001 and **** *P* < 0.0001, Student’s *t-*test.

To understand how MIR99AHG regulates inflammatory responses in macrophages, we examined its location by performing sub-cellular fractionation and analysed the expression of MIR99AHG by RT-qPCR. This analysis revealed that MIR99AHG was predominantly found in the cytoplasm in unstimulated BMDMs but upon IL-4/IL-13 stimulation MIR99AHG translocated to the nucleus (Figure 6A) suggesting that MIR99AHG may exert its biological functions in the nucleus in M2 (IL-4/IL-13) polarized macrophages.

To identify MIR99AHG binding proteins, we performed RNA pulldown assay *in vitro* by incubating transcribed biotinylated MIR99AHG and its antisense control RNA with nuclear extracts from IL-4/IL-13 stimulated and Mtb HN878 infected BMDMs. The associated proteins were pulled down and analysed by mass spectrometry. We identified the 6 most enriched MIR99AHG-binding proteins based on the number of peptides specifically associated with MIRR9AHG (Figure 6B). Among the 6 genes identified, hnRNPA2/B1 had the highest number of peptides associated with MIR99AHG (Figure 6B). To confirm hnRNPA2/B1-MIR99AHG interaction, we performed RNA immunoprecipitation (RIP) of endogenous hnRNPA2/B1 nuclear extracts of BMDMs stimulated with IL-4/IL-13 and infected with Mtb HN878 followed by RT-qPCR analysis and Western blot (Figure 6C and D). These data show that MIR99AHG is translocated to the nucleus upon IL-4/IL-13 stimulation and interacts with hnRNPA2/B1 in macrophages. To assess the impact of MIR99AHG knockdown on gene expression in macrophages, we performed RNA-seq in MIR99AHG knockdown BMDMs infected with Mtb HN878 for 4 and 24 hours (Figure 6E, F). BMDMs were transfected with control and ASO-MIR99AHG for 48 hours prior to Mtb infection. We regarded this time point as 0 hour. BMDMs were then infected with Mtb HN878 for 4 and 24 hours. We compared up and down regulated genes in control treated and ASO-MIR99AHG treated BMDMs at 4 and 24 hours post Mtb (Figure 6E, F). The heatmap shows 61 genes that are constantly up and down regulated at 4 and 24 hours post Mtb infection (Figure 6G). The most downregulated genes in MIR99AHG vs control at 24 hours post Mtb infection include Fbxo8, Galt, Gmds, Patz1 and Znhit6 (Figure 6G). The most upregulated genes in MIR99AHG vs control at 24 hours post Mtb infection include Ckm, Ahsg, Myl1, il12b and Areg (Figure 6G). Some of the upregulated genes that were significantly enriched related to regulation of inflammatory responses, and activation of innate immune response (Figure 6G).

## Discussion

Host-directed drug therapies (HDTs) main purpose is to eliminate Mtb with minimal damage to the host [34]. HDTs include different groups of compounds, such as cytokines, repurposed-drugs that target biologically and clinically relevant checkpoints in anti-Mtb-directed host response pathways [34]. Adjunctive HDTs have the potential to shorten TB treatment duration, prevent antibiotic resistance and reduce lung injury by promoting autophagy, antimicrobials and other macrophage effector mechanisms [35]. Corticosteroids have been used as adjunctive therapy for many inflammatory and disease states [36,37]. The rationale for using corticosteroids in active TB disease involves modulation of inflammatory and apoptotic gene transcription pathways [38]. Corticosteroids as immunoadjuvants to standard TB treatments has proven to be useful in several studies including treatment of HIV/TB coinfection [39–42]. The first therapeutic nucleic acid, DNA-based oligonucleotide, approved by the FDA was Fomivirsen [43]. Recently, the first RNA-based ASO was approved by the FDA [44]. This treatment approach promises to be a growing field as many biopharmaceutical companies are developing RNA interference (RNAi)-based and RNA-based antisense oligonucleotide therapies [45]. The potential of using lncRNAs and miRNAs as adjunctive HDT is a topic that is gaining momentum especially in the context of treating infectious diseases. A handful of miRNA-targeting drugs have entered into clinical trials targeting different diseases [45].

M2 (IL-4/IL-13) polarized macrophages are immune modulators that secrete ornithine and mediate Th2-associated effector functions [46]. Mtb neutralizes microbicidal effectors to promote M2 (IL-4/IL-13) polarization state to promote persistence and survival in macrophages [46]. An effective immune defence against intracellular pathogens is dependent on a wide transcriptional program in macrophages and other immune cells [47]. While the role of miRNAs in the regulation host inflammatory responses has been well studied [48], the role of long non-coding RNAs in macrophages and during Mtb infection is still poorly understood.

In this study, we demonstrate that the long intergenic non-coding RNA, MIR99AHG is highly upregulated in M2 (IL-4/IL-13) polarized macrophages. We analyzed the expression of MIR99AHG in murine macrophages infected with either hypervirulent Mtb HN878, virulent H37Rv, avirulent Mtb H37Ra and *Mycobacterium bovis* BCG strains. MIR99AHG was downregulated by Mtb HN878, Mtb H37Rv and Mtb H37Ra infection but upregulated in *Mycobacterium bovis* BCG infected macrophages. Similarly, long non-coding RNA, HOTAIR, was dowregulated by Mtb H37Rv but upregulated by Mtb H37Ra [49]. Using antisense therapy (ASOs), we gained insights into the biological functions of MIR99AHG at both cellular level and organismal level. Knockdown of MIR99AHG by ASOs reduced intracellular Mtb growth in mouse and human macrophages, induced apoptosis and inhibited necrosis. Knockdown of MIR99AHG by ASOs reduced proinflammatory cytokines such as IL-6 and IL-1β. *In vivo* inhibition of MIR99AHG by ASOs reduced mycobacterial burden in the lung and spleen and reduced pro-inflammatory responses in lung macrophages. We show that MIR99AHG interacts with hnRNPA2/B1. We further demonstrate that MIR99AHG is translocated to the nucleus following IL-4/IL-13 stimulation.

MIR99AHG was previously identified and referred to as MONC [50]. MIR99AHG was reported to be spliced into a 710-nucleotide lincRNA, where intron 6 of MONC produces three miRNAs *MIR99A, LET7C* and *MIR125B2* [50]. The knockdown of MIR99AHG by shRNAs in acute megakaryoblastic leukemia (AMKL) cell lines reduced leukemic growth [50]. Similarly to our study, upon MIR99AHG knockdown by ASOs, apoptosis was increased in AMKL cell lines [50]. Furthermore, MONC was predominantly located in the nucleus [50]. In a recenty published paper MIR99AHG was downregulated in lung adenocarcinoma tissues [51]. Knockdown of MIR99AHG suppressed the proliferation and metastasis and increased autophagy *in vitro* and *in vivo* in H1299 or A549 cells [51].

IL-4 signals through two different receptor complexes, type I receptor (IL-4Rα/cγ chain) andtype II receptor (IL-13Rα1) [52,53]. Since MIR99AHG is upregulated by IL-4/IL-13, we examined its expression in IL-4Rα^-/-^ BMDMs and in lung homogenate of naïve and Mtb HN878 infected mice. We observed that MIR99AHG expression was mediated by the IL-4Rα signalling pathway in M2 (IL-4/IL-13) polarized macrophages (Figure S1). Interestingly, we observed high MIR99AHG expression in lung homogenates of naïve, non-infected mice (Figure 1H). This high MIR99AHG expression could potentially be explained by the high levels of IL-4 detected in lungs of naïve BALB/c mice [54]. Apoptosis is an important innate host defence mechanism to control mycobacterial growth within macrophages [55]. It has been reported that virulent strains of Mtb inhibit apoptosis [56], while avirulent Mtb strains induce apoptosis in macrophages resulting in the inhibition of mycobacterial growth [57]. Furthermore, virulent Mtb infection causes necrosis in macrophages that allows mycobacterial dissemination [56]. Mtb has been shown to be able to survive in necrotic macrophages before escaping [58]. Corticosteroids which have been used as HDT were shown to inhibit the necrotic cell death of Mtb infected cells through the dephosphorylation of p38 MAPK [59]. Bax is a known pro-apoptotic marker, and this is supported by many previous studies who have measured the expression of Bax as a pro-apoptotic marker [60–65]. From our findings we show that the knockdown of MIR99AHG by ASOs increases apoptosis but reduces necrosis in Mtb HN878 infected macrophages.

The secretion of pro-inflammatory cytokines is crucially important in the protection of the host against Mtb infection. To control mycobacterial replication, cytokines such as IFNγ, TNFα, IL-6, IL-1β are required for an effective host immune response against Mtb [66]. We examined whether knocking down of MIR99AHG would modulate pro-inflammatory cytokine production in macrophages. Knockdown of MIR99AHG by ASOs significantly reduced the production of IL-6 and IL-1β in M2 (IL-4/IL-13) polarised and Mtb HN878 infected macrophages suggesting that MIR99AHG may be a positive regulator of pro-inflammatory cytokine responses. A number of long non-coding RNAs have been reported to regulate host inflammation or act as “brakes” in controlling excessive inflammation [16,18,67,68]. Our results suggest that MIR99AHG plays an immunomodulatory role as a potential regulator of macrophage polarization and inflammation promoting Mtb growth and persistence in macrophages.

The role of lncRNAs in cancer has been well studied, however there is limited data available on characterized lncRNAs in a TB disease model. Recent studies have shown strong evidence on the role of lncRNAs in the modulation of innate and adaptive immune response [16,18,67]. Findings from animal models, using systemic administration of an ASOs, serve as a translational proof of concept for therapeutic interventions targeting lncRNAs [69]. In this study, we provide evidence for MIR99AHG modulating host immunity to Mtb infection. We explored the role of MIR99AHG in a loss of function approach using ASO therapy *in vivo*. Mice treated with MIR99AHG ASOs were more protected against TB disease exhibiting reduced lung inflammation. In histological lung sections, Caspase-3 staining was significantly increased in the MIR99AHG ASO treated group suggesting increased apoptosis. Moreover, ASO treated murine macrophages displayed enhanced apoptosis and increased Bax Mrna expression following Mtb HN878 infection further supporting a possible mechanism for the observed reduced mycobacterial growth. NOS2 mRNA expression in lung macrophages was significantly reduced in ASO-MIR99AHG treated mice suggesting that the reduced mycobacterial burden was independent of NOS2 effector functions. Nitric oxide has been identified to be a key player in controlling *M. tuberculosis* infection [70]. We show for the first time that treatment with MIR99AHG ASOs significantly reduced the pathogenesis of Mtb HN878 infection, suggesting that MIRR9AHG could be repurposed for host-directed therapy against TB.

The engagement of toll like receptors (TLRs) elicits signalling pathways that activate inflammatory genes and many long noncoding RNAs have been shown to be induced by TLRs [16,18,67,68]. Long noncoding RNAs have emerged as new regulators of inflammatory mediators in the immune system. In this study we show the response of mRNA expression of MIR99AHG was significantly upregulated in BMDMs exposed CpG (TLR-9) at 4 hours post stimulation suggesting that MIR99AHG upregulation is signalled through TLR-9 and MIR99AHG downregulation is signalled through TLR-2/4 (Figure 2A-C). Similarly, THRIL (TNF-α hnRNPL Related Immunoregulatory Long non-coding RNA) was shown to be downregulated following TLR-2 stimulation in THP-1 cells [17]. In contrast to THRIL, lincRNA-Cox2 was highly upregulated by TLR-2 and TLR-4 [16] in BMDMs. NKILA is another lncRNA upregulated by TLR-4 stimulation in MDA-MB-231 in breast cancer cells [71]. There have been no published lncRNAs reported to be upregulated or downregulated by TLR-9. Many functions of lncRNAs are mediated via control of TLR signalling and altered expression and activities of lncRNAs have been implicated in the pathogenesis of infectious and inflammatory diseases [72].

Our study demonstrated that MIR99AHG interacted with hnRNPA2/B1. Interestingly, hnRNPA2/B1 has been shown to interact with *lincRNA-COX2* to regulate immune response genes during inflammatory responses [16]. Macrophages lacking the expression of hnRNPA2/B1 by shRNA resulted in enhanced levels of *Ccl5, Trl7, Il6, Il1b, Stat1 and IκB* following LPS and Pam3Csk4 stimulation [16]. In our study we showed that knockdown of MIR99AHG by ASOs reduced *Il6, Il1b* and *Nos2* mRNA expression. The hnRNP family acts as mediators of lncRNA-induced transcriptional gene repression [73,74]. The interaction of lncRNA-p53 with its protein binding partner hnRNP-K is essential for the genomic localization of hnRNP-K at repressed genes and the regulation of p53 mediated apoptosis [74]. hnRNPs are important functional partners for many additional lncRNAs. These include *lincRNA-EPS* which interacts with hnRNPL [14]. *lincRNA-p21* [74] and *Xist* [73] have been identified as binding partners of hnRNPK and hnRNPU, respectively. hnRNPs are well known to be involved in mRNA biogenesis. In addition, the role of hnRNPA2/B1 in transcriptional regulation of gene expression is emerging. For example, hnRNPA2/B1 interacts with small activating dsRNA to induce transcriptional activation, is required [75]. Moreover, hnRNPA2/B1 is required for smooth muscle cell (SMC) differentiation gene expression. hnRNPA2/B1 and promotes neural crest cell migration [76]. The identification of hnRNPA2/B1 as a functional binding partner of MIR99AHG adds further support to the role of hnRNPs in transcriptional regulation. The MIR99AHG-hnRNPA2/B1 interaction observed in this manuscript is of importantance, further experiments need to be performed to understand the mechanism and significance of this interaction on target genes to fully substantiate this interaction. In addition, further work can be performed such as mapping of the binding domains of the interacting partners and mutational analysis to understand the consequences of abrogating the interaction.

Our RT-qPCR results on purified RNA from the nucleus and cytoplasm compartments showed a nuclear localization of MIR99AHG upon IL-4/IL-13 stimulation. It is most likely that lncRNAs play an important role in the organization of nuclear domains [77]. In the nucleus, lncRNAs can act in *cis* or *trans*, for example *Morrbid* interacts with PRC2 to repress transcription of the neighbouring gene, Bim (*Bcl2l11*) [78]. A lncRNA can interact with its protein partner as a guide (lincRNA:hnRNPL) [14], scaffold (RMRP interaction with DDX5 and RORγt) [79] or decoy molecule (Lethe:NF-κB p65) [80] to mediate its molecular functions. Cellular localization of lncRNAs can help with studies involving functions and mechanisms of action. Further studies in identifying the exact subnuclear areas and DNA target sequences of MIR99AHG still need to be performed.

In conclusion, this study characterizes, for the first time, the functional role of MIR99AHG during Mtb HN878 infection. MIR99AHG plays an important role in the regulation of inflammatory genes and promotes Mtb growth in macrophages. In Mtb HN878 infected mice and macrophages, MIR99AHG promotes the intracellular survival of Mtb possibly through favouring macrophage necrosis. MIR99AHG, as adjunct to existing antibiotics could potentially become a possible target for host-directed drug therapy for TB.

## Supplementary Material

Supplementary figures

Supplementary material
