# Supplementary figures and images for: Host-directed targeting of lincRNA-MIR99AHG suppresses intracellular growth of Mycobacterium tuberculosis
Source: Nucleic Acid Ther. Author manuscript; Available in PMC 2022 Oct 22. (PMC7613730; doi:10.1089/nat.2022.0009)

## Slide 1
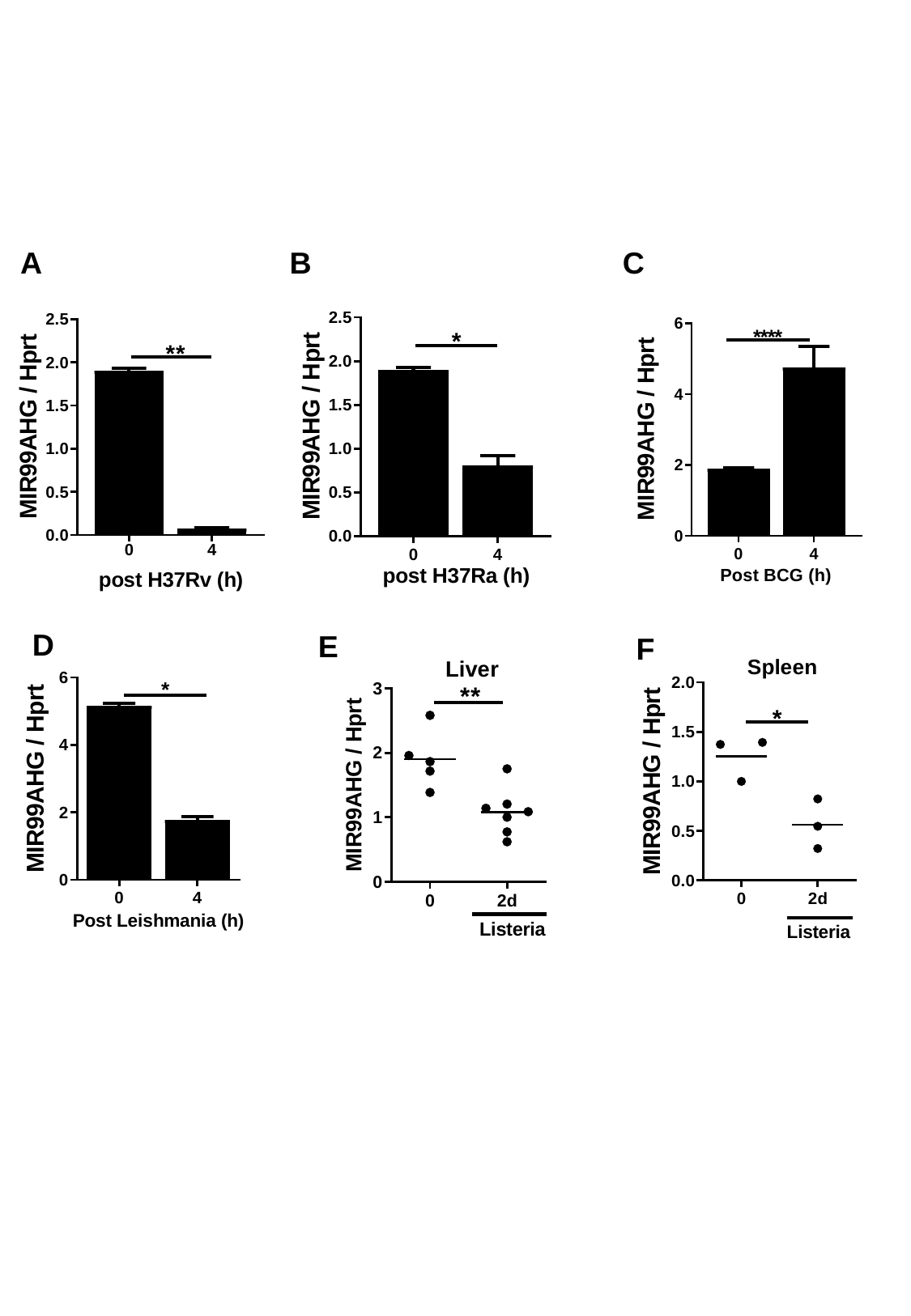

## Slide 2
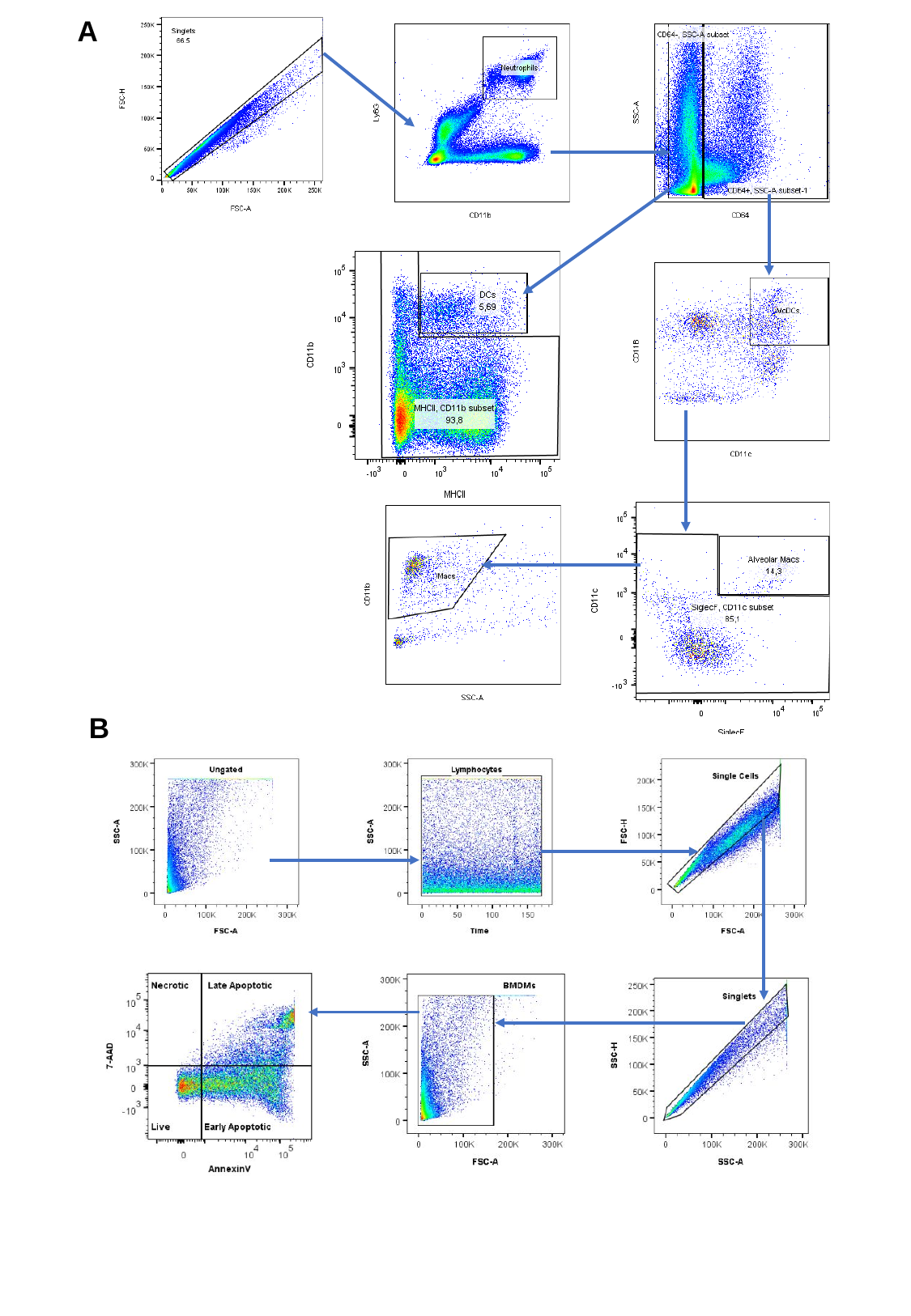

## Slide 3
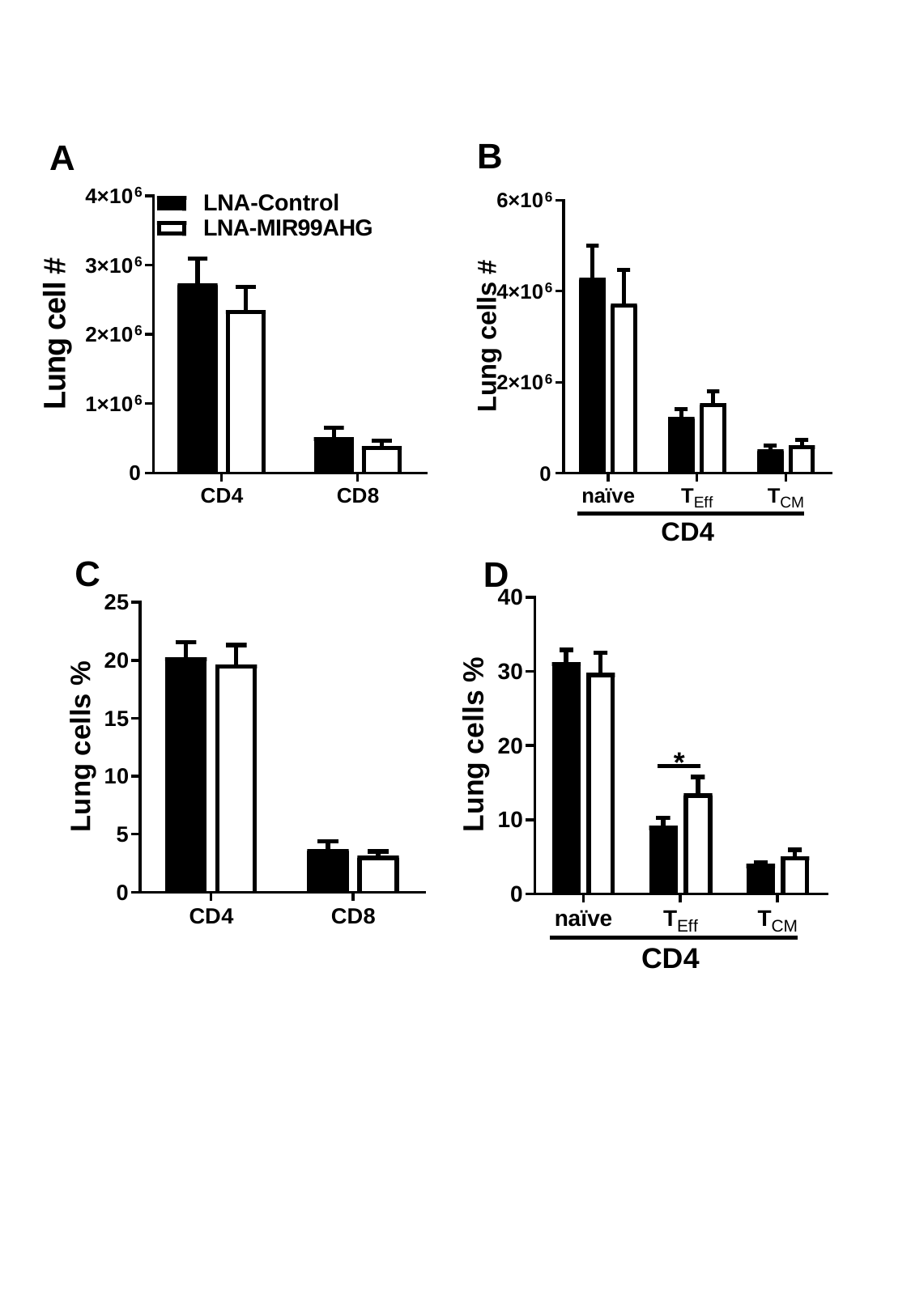

## Slide 4
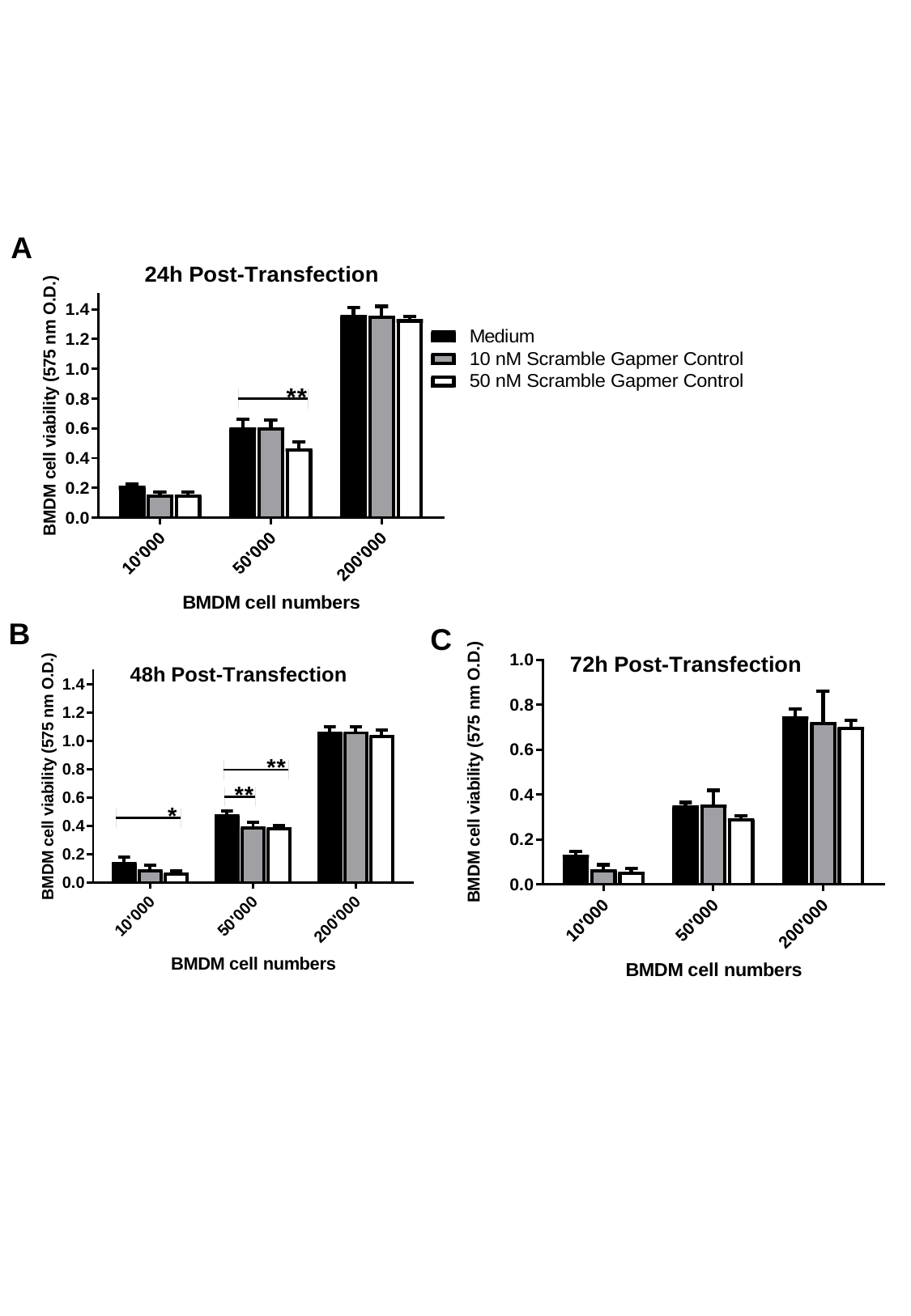

Supplement: Supplementary figures [file EMS155541-supplement-Supplementary_figures.pptx]
