# Supplementary material for: Host-directed targeting of lincRNA-MIR99AHG suppresses intracellular growth of Mycobacterium tuberculosis
Source: Nucleic Acid Ther. Author manuscript; Available in PMC 2022 Oct 22. (PMC7613730; doi:10.1089/nat.2022.0009)
Supplement: Supplementary material [file EMS155541-supplement-Supplementary_material.docx]

**Figure S1. MIR99AHG mRNA expression following Mtb H37Rv, *Mycobacterium bovis* BCG, *Leishmania mexicana* and  *Listeria monocytogenes infection.*** (**A-D**) MIR99AHG mRNA expression by RT-qPCR in BMDMs pre-stimulated with IL-4/IL-13 for 4 hours and infected with Mtb H37Rv, H37Ra , *Mycobacterium bovis* BCG and *Leishmania mexicana* M379 strain for 4 hours. (**E, F**) MIR99AHG mRNA expression by RT-qPCR in whole liver (n = 5 mice) and spleen (n = 3 mice) from wild-type mice infected with *Listeria monocytogenes* (2x105 CFU/mouse) at 2 dpi. Data are expressed as mean ± SD of triplicates. Data shown (A, B, C, E) is representative of one experiment. Data (D) are representative of three independent experiments. P values represented as, * *P* < 0.05, ** *P* < 0.01, and **** *P* < 0.0001; Student’s *t*-test.

**Figure S2. Gating strategies of lung lyeloid sorted cells and apoptotic cells.** (**A, B**) Gating strategies of lung myeloid sorted cell populations and apoptotic cells. Data are expressed as mean ± SD of triplicates. Data are representative of three independent experiments. *P* values represented as, **P* < 0.05 and ***P* < 0.01, Student’s *t*-test.

**Figure S3. T cell lung cell percentages and numbers.** BALB/c mice (n= 6 mice/group) were ASO treated with locked nucleic acid control and ASO-MIR99AHG at 10 mg/kg for alternative days up to day 14. Mice were then infected with intranasally with 100 CFU/mouse of Mtb HN878 and killed at 3 weeks post Mtb infection. Total lung numbers of **(A)** CD3^+^CD4^+^, CD3^+^CD8^+^ T cells; **(B)** CD44^low^CD62L^high^ naïve CD4^+^, effector CD44^high^CD62L^low^ CD4^+^ T cells and central memory CD44^high^CD62L^high^ CD4+. Percentages of lung **(C)** CD3^+^CD4^+^, CD3^+^CD8^+^ T cells; **(D)** CD44^low^CD62L^high^ naïve CD4^+^, effector CD44^high^CD62L^low^ CD4^+^ T cells and central memory CD44^high^CD62L^high^ CD4+.

**Figure S4. Dose response on BMDM cell viability following transfection with Scramble Gapmer negative control.** BMDMs were seeded with different cell numbers (10’000, 50’000 and 200’000 cells) on 96-well cell culture plate and then transfected with either 10nM or 50nM of the Scramble Gapmer negative control. Cell viability was measured in BMDMs by Cell Titer Blue at **(A)** 24, **(B)** 48 and **(C)** 72 hours post transfection. Data are representative of three independent experiments. Data are expressed as mean ± SD of triplicates. P values represented as, **P* < 0.05, ***P* < 0.01; Student’s *t*-test.
